# Supplementary material for: Robust thermoelastic microactuator based on an organic molecular crystal
Source: Nat Commun. 2019 Oct 8;10:4573. doi: 10.1038/s41467-019-12601-y (PMC6783412; doi:10.1038/s41467-019-12601-y)
Supplement: Supplementary file 1 — Supplementary Information [file 41467_2019_12601_MOESM1_ESM.pdf]

Supplementary Information for

Robust thermo-elastic micro-actuator based on an organic molecular crystal

Yulong Duan[1], Sergey Semin[1], Paul Tinnemans[1], Herma Cuppen[1], Jialiang Xu[2]*, Theo Rasing[1]*.

[1]Radboud University Nijmegen, Institute for Molecules and Materials, Heyendaalseweg 135, 6525 AJ, Nijmegen, the Netherlands.

[2]School of Materials Science and Engineering, Nankai University, Tongyan Road 38, Tianjin 300350, P.R. China.

Corresponding authors: Jialiang Xu (jialiang.xu@nankai.edu.cn) Theo Rasing (th.rasing@science.ru.nl)

This file includes:

Supplementary Figures 1-8
Supplementary Table 1
Supplementary References 1-3

Other Supplementary Materials for this manuscript include the following:

Supplementary Movies 1 to 5

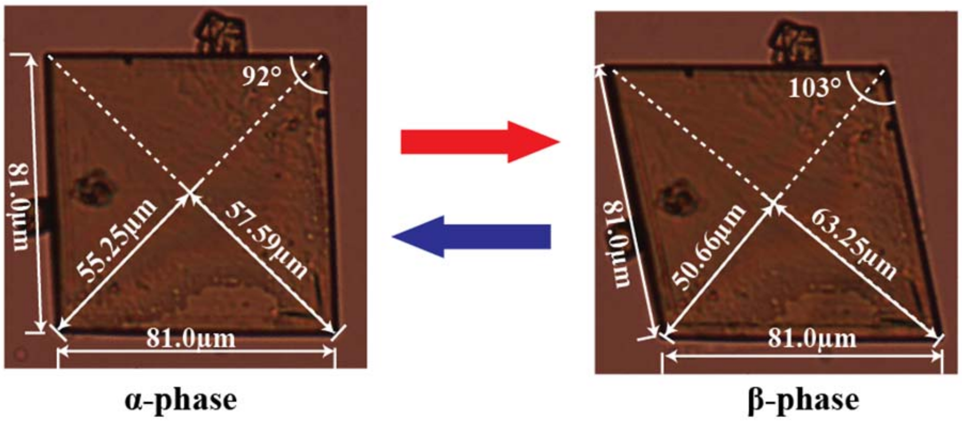

Supplementary Figure 1. Optical microscope images of the in-plane crystal shape change during the phase transition. This crystal has equal side lengths, so the diagonal directions are along the in-plane crystal axes. During the phase transition, the side lengths stay the same, although the diagonal directions undergo large anisotropic expansions. At around 170 °C, right before the phase transition, the corner angle of the α-phase can be calculated from: 2arctan (57.59μm/55.25μm) ≈ 92°, which coincides to that calculated from the in-plane unit cell: 2arctan (7.27 Å /6.90 Å) ≈ 92°. At around 180 °C, right after the phase transition, the corner angle of the β-phase can be calculated from: 2arctan (63.25μm/50.66μm) ≈ 103°, which also coincides to that calculated from the in-plane unit cell: 2arctan (7.77 Å /6.25 Å) ≈ 103°.

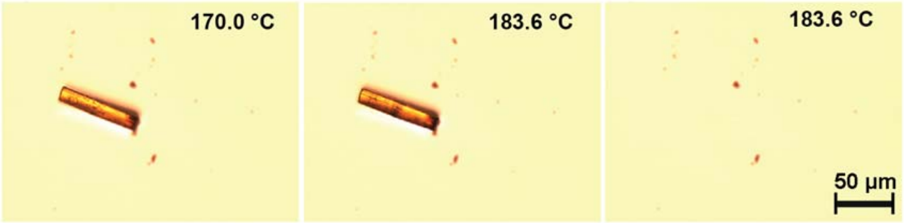

Supplementary Figure 2. Crystal jumping during the phase transition. The crystal was placed standing sideways on the substrate. Because of the large in-plane shear deformation, the crystal jumps out of the field of view by slightly increasing the temperature at the phase transition point (183.6 °C).

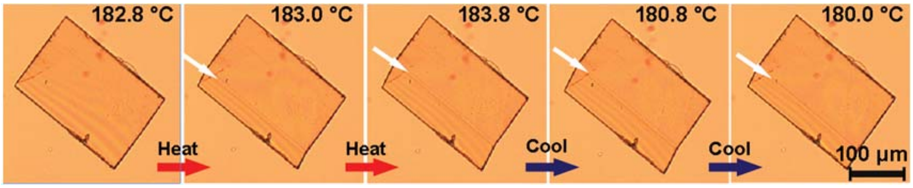

Supplementary Figure 3. Phase boundary position controlled by tuning the temperature. The crystal was heated to 183.8 °C and then cooled to 180.0 °C. The phase boundary, indicated by the arrow, initially progressed and then came to halt upon cooling. Cooling further below 180.8 °C, lead to retracing of the phase boundary.

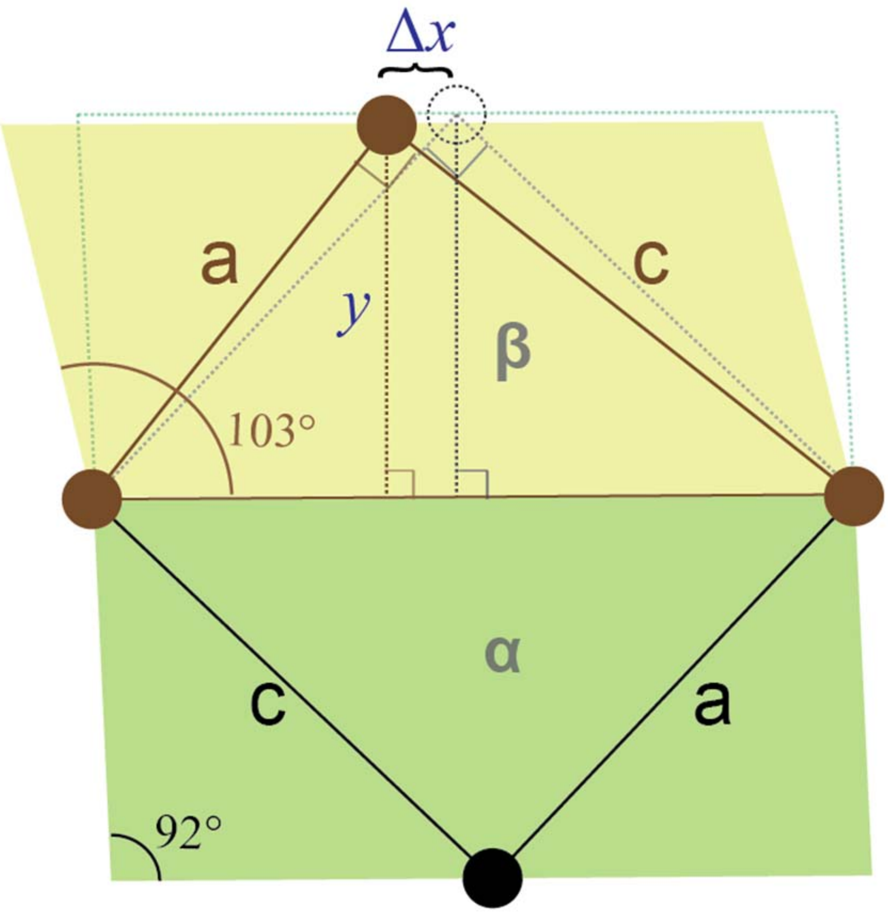

Supplementary Figure 4. Calculation of the induced shear strain during the phase transition. The crystallographic a-axis shrinks 10% and the c-axis expands 7% when the crystal is heated from 177 °C to 187 °C, but the b-axis stays almost the same. The diagonal length of the in-plane lattices also does not change: $\sqrt{a_{170°\mathrm{C}}^2 + c_{170°\mathrm{C}}^2} = \sqrt{a_{178°\mathrm{C}}^2 + c_{178°\mathrm{C}}^2} = 10$ Å. The shear strain of the crystal during the phase transition is calculated from the in-plane lattice parameters of the two phases. The relative displacement (Δx) along the phase boundary (the in-plane lattice diagonals) is as large as 0.61Å for each step. The cell lengths of a, c are 6.9 Å and 7.27 Å in the α-phase while they are 6.25 Å and 7.77Å in the β-phase, respectively. The length y is 4.85 Å in the β-phase, calculated from the side lengths of the grey right triangle. Therefore, the shear strain is calculated as $\frac{\Delta x}{y} = 0.18$. The colored shapes mark the macroscopic crystal shape of the α-phase (green) and β-phase (yellow). The corner angles of the crystal shape are calculated from the lattice parameters, which are corresponding well with the measured values.

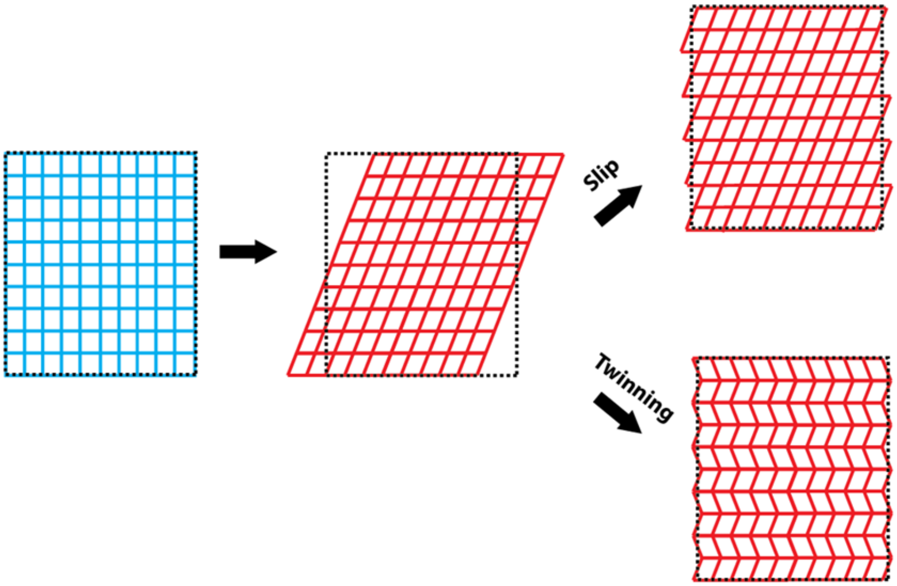

Supplementary Figure 5. Lattice invariant shear in martensitic phase transitions. To minimize the strain energy during the shape change, the boundary plane between the two phases should be undistorted and not rotate (invariant phase boundary). For general martensitic phase transitions, the phase boundary is not invariant due to the lattice parameter changes in the plane of phase boundary (for example the blue lattice changes to the red lattice). A lattice invariant shear is needed to keep an invariant phase boundary (the black dashed frames). There are two ways to achieve a lattice invariant shear, twinning and slip, which will generate microstructures in metals but can easily cause breakages in organic crystals. During the phase transition between the α- and β-phase of 4-DBpFO, the variance of the phase boundary (**{101}** plane) is very small, as both the length of the *b*-axis and the diagonal length of the lattice in the **{010}** plane stay almost the same. The excellent reversibility of the shape change in 4-DBpFO may originate from the invariance of the phase boundary, and an extra lattice invariant shear is not needed during the deformation.

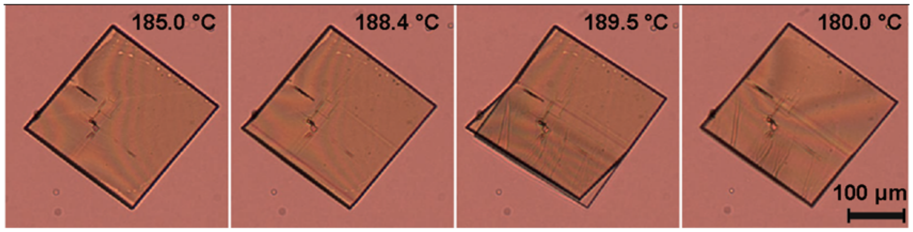

Supplementary Figure 6. Crystal splitting in a large crystal during the phase transition. A crystal splits in the transformed phase perpendicular to the phase boundary (3rd image from the left). The reason is probably the relative sliding between the layers during the migration of the phase boundary. By cooling the crystal back to the *a*-phase (4th picture from the left), the shape is recovered though the damage due to the splitting is not.

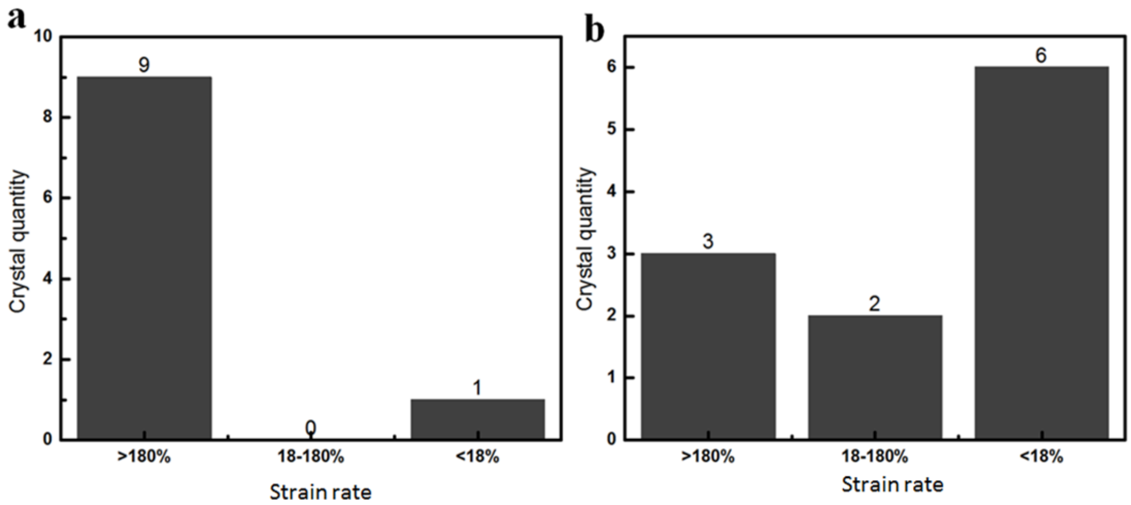

Supplementary Figure 7. Distribution of the strain rate for different crystal sizes. **(a)** Crystals with top surface smaller than 100 × 100 μm^2. **(b)** Crystals with top surface larger than 100 × 100 μm^2. Note that the crystal used to kick the glass bead shown in the main text has a strain rate larger than 180%.

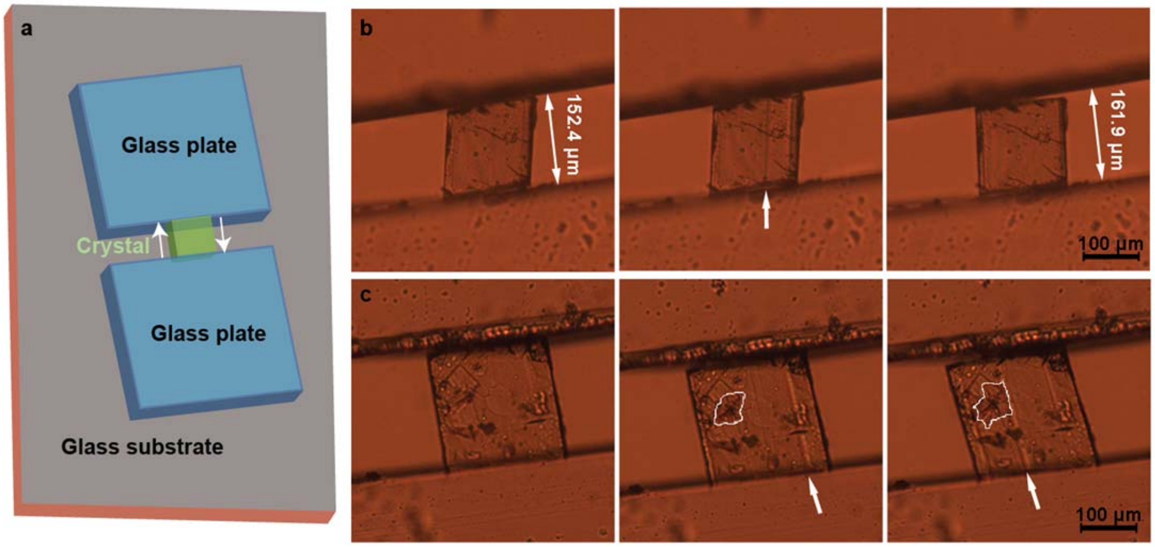

Supplementary Figure 8. Demonstration of actuator force and damage threshold. The maximum pushing force of the crystals was tested by positioning a crystal between two parallel glass plates. **(a)** Schematic diagram of experiment. Sample is placed between two glass plates on a flat glass substrate. The glass plates are moved apart by the crystal at the phase transition temperature and the maximum pushing force was estimated by the maximum static friction between the glass plates and the glass substrate. The arrows show the direction of the thermal induced crystal shear. (**b)** Each glass plate weighs 60 mg, which is ~3× 10^4 times heavier than the crystal (160×160×60 μm). The glass plates were pushed apart by the crystal during the shape deformation and the crystal was not destroyed or cracked during the shape change. The force generated by the shape change was estimated to be at least 530 μN. (**c)** Each glass plate weighs 260 mg, which is ~10^5 heavier than the crystal (200×200×50 μm). The force generated by the shape change was estimated to be at least 2300 μN. The glass plates can be pushed apart by the crystal, but the crystal was 'damaged' during the shape change. This destruction is a result of the disruption of the cooperative molecular movement. An incoherent phase boundary can be observed during the phase transition (irregular white lines) in addition to the coherent phase boundaries (line white arrows). Pictures in A and B were extracted from supplementary movie S4 and S5, respectively. Notice that the crystal cannot be deformed by applying a stress as those organosuperelastic crystals studied by Takamizawa *et al*[1].

Supplementary Table 1. The performance of some typical actuators.

	Strain (%)	Strain rate (%/s)	Work density
**4-DBPFO crystal**	18	>180	270 J/kg 87.9 KJ/m^3
**Biological muscle**[2]	20-40	>50	8-40 KJ/m^3
**Artificial muscle (Nylon-6,6)** [2]	4-33		2.48KJ/kg (84 MPa)
**Shape-Memory Alloy**[2]	<8.5	<300	<10 mJ/m^3
**Piezoelectric actuators**[2]	<380		<3.5 mJ/m^3
**thermosalient crystals (PHA)**[3]	0.3-1.6		

Supplementary References

1. Takamizawa, S., Takasaki, Y., Sasaki, T. & Ozaki, N. Superplasticity in an organic crystal. *Nat. Commun.* **9**, 3984 (2018).
2. Mirvakili, S. M. & Hunter, I. W. Artificial Muscles: Mechanisms, Applications, and Challenges. *Adv. Mater.* **30**, 1704407 (2018).
3. Khalil, A., Karothu, D. P. & Naumov, P. Direct Quantification of Rapid and Efficient Single-Stroke Actuation by a Martensitic Transition in a Thermosalient Crystal. *J. Am. Chem. Soc.* **141**, 3371-3375 (2019).
