# Supplementary material for: Robust thermoelastic microactuator based on an organic molecular crystal
Source: Nat Commun. 2019 Oct 8;10:4573. doi: 10.1038/s41467-019-12601-y (PMC6783412; doi:10.1038/s41467-019-12601-y)
Supplement: Supplementary file 3 — Description of Additional Supplementary Files [file 41467_2019_12601_MOESM3_ESM.pdf]

Description of Additional Supplementary Files

File Name: Supplementary Movie 1
Description: The movie shows the shape deformation of a crystal during the phase transition by heating. The phase transition proceeds by the migration of a coherent phase boundary, which can be clearly seen during the shape change. The heating speed was 3 °C/min.

File Name: Supplementary Movie 2
Description: Movie of MD simulation of the phase transition corresponding to the data in Fig. 4. The phase transition is triggered by a change in the dihedral angle of the phenyl rings. These phenyl rings are displayed thicker here and are color coded according to their dihedral angle (blue to red). A clear nucleation center can be observed. The real timescale of the movie is about 27.5 ps.

File Name: Supplementary Movie 3
Description: The movie shows the 'kick' of a glass bead by a crystal during the shape change. The video was captured by using a CCD camera with 30 frames per second. The speed of the movie is slowed down to see the process. The real timescale of the video is about 0.12 sec.

File Name: Supplementary Movie 4
Description: The movie shows the displacement of a glass plate by the shape change of the crystal. The crystal was positioned between two glass plates. The heavy glass plates can be moved a small distance due to the shear deformation of the crystal without breaking it. The mass of every glass sheet is around 60 mg, which is 104 times heavier than the crystal (160×160×60 µm). The real timescale of the video is about 30 sec.

File Name: Supplementary Movie 5
Description: The movie shows that an incoherent phase boundary can be clearly seen during the phase transition, when the crystal was positioned between much heavier glass plates. Although a temperature increase can still make the crystal to undergo the shape change, an incoherent phase boundary appeared in addition to the coherent phase boundaries, which indicates that the cooperative molecular movements during the phase transition were distorted. The mass of every glass plate is around 260 mg, which is 105 times heavier than the crystal (200×200×50 µm). The real timescale of the video is about 30 sec.
